# Chromosomal mapping of rDNAs and H3 histone sequences in the grasshopper *rhammatocerus brasiliensis *(acrididae, gomphocerinae): extensive chromosomal dispersion and co-localization of 5S rDNA/H3 histone clusters in the A complement and B chromosome

**DOI:** 10.1186/1755-8166-4-24

**Published:** 2011-11-10

**Authors:** Nathalia L Oliveira, Diogo C Cabral-de-Mello, Marília F Rocha, Vilma Loreto, Cesar Martins, Rita C Moura

**Affiliations:** 1 Departamento de Biologia, Instituto de Ciências Biológicas, UPE - Universidade de Pernambuco, Recife, Pernambuco, Brazil; 2 Departamento de Biologia, Instituto de Biociências, UNESP - Universidade Estadual Paulista, Rio Claro, São Paulo, Brazil; 3 Departamento de Genética, Centro de Ciências Biológicas, UFPE - Universidade Federal de Pernambuco, Recife, Pernambuco, Brazil; 4 Departamento de Morfologia, Instituto de Biociências, UNESP - Universidade Estadual Paulista, Botucatu, São Paulo, Brazil

**Keywords:** evolution, genome, cytogenetics, multigene family

## Abstract

**Background:**

Supernumerary B chromosomes occur in addition to standard karyotype and have been described in about 15% of eukaryotes, being the repetitive DNAs the major component of these chromosomes, including in some cases the presence of multigene families. To advance in the understanding of chromosomal organization of multigene families and B chromosome structure and evolution, the distribution of rRNA and H3 histone genes were analyzed in the standard karyotype and B chromosome of three populations of the grasshopper *Rhammatocerus brasiliensis*.

**Results:**

The location of major rDNA was coincident with the previous analysis for this species. On the other hand, the 5S rDNA mapped in almost all chromosomes of the standard complement (except in the pair 11) and in the B chromosome, showing a distinct result from other populations previously analyzed. Besides the spreading of 5S rDNA in the genome of *R. brasiliensis *it was also observed multiple sites for H3 histone genes, being located in the same chromosomal regions of 5S rDNAs, including the presence of the H3 gene in the B chromosome.

**Conclusions:**

Due to the intense spreading of 5S rRNA and H3 histone genes in the genome of *R. brasiliensis*, their chromosomal distribution was not informative in the clarification of the origin of B elements. Our results indicate a linked organization for the 5S rRNA and H3 histone multigene families investigated in *R. brasiliensis*, reinforcing previous data concerning the association of both genes in some insect groups. The present findings contribute to understanding the organization/evolution of multigene families in the insect genomes.

## Background

In eukaryotes the transcribing sequences for ribosomal RNAs (rRNAs) are organized in two multigenic families tandemly arrayed in the genomes. The major one (45S rDNA) transcribes the 18S, 5.8S and 28S rRNAs and the minor one (5S rDNA) is responsible for the transcription of the 5S rRNA [1]. Concerning the histone gene sequences, they may be arranged in tandemly repeated clusters composed by intronless genes that codes for H1, H2A, H2B, H3 and H4 histone proteins spaced by noncoding DNA, although some variation has been reported for this organization [2,3].

Due to the clustered organization of multigene families and other repetitive DNAs they have been an important source as chromosomal marker for analysis of karyotypic evolution, genomic structure and origin and evolution of B chromosomes in animals. In grasshoppers the mapping of repeated elements is primarily concentrated in analysis of number and location of rDNAs and histone genes, and in the lesser extend satellite DNA (satDNAs) [4-9]. Concerning multigene families in Acrididae the major rDNA was mapped in 53 species, the histone genes were located in chromosomes of 39 species and the 5S rDNA distribution was described in about 30 species [4-7,9,10]. The mapping of these sequences together with the satDNA has been an important tool for understanding several aspects of the chromosome biology of acridid grasshopers [4,9,10].

Supernumerary B chromosomes occur in addition to standard karyotype and have been described in about 15% of eukaryotes. Some studies have been conducted on B elements, concerning their distribution, frequency in populations, structure and origin, effects in standard complement and transmission [11,12]. The B chromosomes are relatively well studied in grasshoppers, as for example in *Eyprepocnemis plorans *and *Locusta migratoria*. In this group variability for B chromosomes related to heterochromatin patterns, satellite DNAs organization and multigene families has been reported [4,9,10,12-14].

To advance in the understanding of chromosomal organization of multigene families (rDNAs and histone genes) among grasshoppers related to A complement and B chromosomes, we investigated here three populations of the species *Rhammatocerus brasiliensis *(Acrididae, Gomphocerinae) that presents a karyotype composed of 2n = 23, X0, acrocentric chromosomes in males, and bears a distinct B chromosome [10].

## Methods

A total of 284 adult males of *R. brasiliensis *were collected in three distinct populations, Serra Talhada (07°59' 31'' S 38°17' 54'' W), 80 individuals; Surubim (07°49' 59'' S 35°45' 17'' W), 147 individuals; and from Vitória de Santo Antão (08°07' 05'' S 35°17' 29'' W), 57 individuals, Pernambuco State, Brazil. The animals were collected in the wild according to Brazilian laws for environmental protection (wild collection permit, MMA/IBAMA/SISBIO n. 2376-1). The experimental research on animals was conducted according to the international guidelines followed by São Paulo State University (Protocol no. 35/08 - CEEAA/IBB/UNESP). All animals were studied under conventional staining to estimate the B chromosome presence and frequency. For molecular cytogenetic analysis DNA and chromosomal spreads were obtained from ten individuals collected in Serra Talhada, six from Surubim and four from Vitória de Santo Antão. The genomic DNA of individuals without B chromosomes was extracted using the phenol-chloroform procedure described by Sambrook and Russel [15]. Meiotic chromosomes were obtained from testicular cells previously fixed in Carnoy (3:1 ethanol: acetic acid) and stored in freezer. The slides were prepared by classic squashing of testicular follicles technique using a drop of acetic acid (50%). Slides used for fluorescence *in situ *hybridization (FISH) analysis were prepared in 45% acetic acid and coverslips were removed after freezing the preparations by immersing in liquid nitrogen for a few seconds.

Partial sequences of 5S rRNA and histone H3 genes were amplified by PCR of genomic DNA from *R. brasiliensis *using the primers designated by Loreto et al. [10] and Cabral-de-Mello et al. [16], Sca5SF (5'-AAC GAC CAT ACC ACG CTG AA-3'), Sca5SR (5'-AAG CGG TCC CCC ATC TAA GT-3'), ScaH3F (5'-GGC NMG NAC NAA RCA RAC-3') and ScaH3R (5'-TGD ATR TCY TTN GGC ATD AT-3'). The 18S rDNA sequence was obtained from a cloned fragment previously isolated from the genome of *Dichotomius semisquamosus *[16].

The plasmid containing the 18S rRNA gene and the PCR product from histone H3 gene were labeled by nick translation using biotin-14-dATP (Invitrogen, San Diego, CA, USA). For simultaneous hybridization the 5S rDNA was labeled using digoxigenin-11-dUTP (Roche, Mannheim, Germany). The FISH procedures were performed according to the modifications of Cabral-de-Mello et al. [16]. The preparations were counterstained using the 4', 6-diamidine-2'-phenylindole dihydrochloride (DAPI) and mounted in Vectashield (Vector, Burlingame, CA, USA). Images were captured with the Olympus DP71 digital camera coupled to a BX61 Olympus microscope and were optimized for brightness and contrast using Adobe Photoshop CS2. The chromosomal morphology was defined using the description by Levan et al. [17].

## Results

All populations of *R. brasiliensis *showed 2n = 23, X0 with acrocentric chromosomes (males), organized into three size-groups: three large (L1-L3), five medium (M4-M8) and three small (S9-S11). The X chromosome was identified as medium element and the B chromosome, when present, showed similar size to the X, as described by Loreto et al. [10,18]. A total of 26 individuals with B chromosome was observed in the total sample, being six from Serra Talhada, 11 from Surubim and nine from Vitória de Santo Antão, corresponding to 7, 5%, 7, 48% and 15, 79% of each sample, respectively [Loreto et al. *in preparation*].

**Figure 1:**
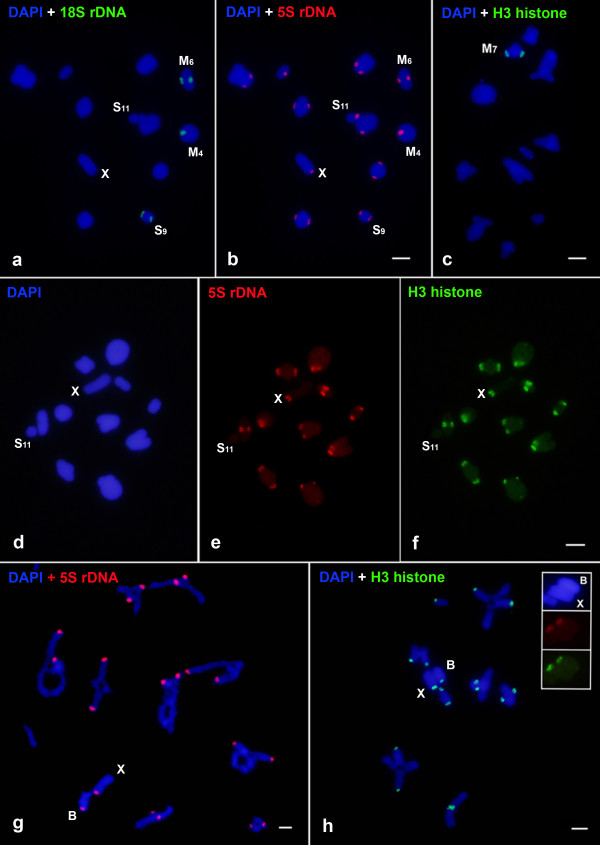
**Fluorescence *in situ *hybridization of 18S rRNA, 5S rRNA and H3 histone gene sequences in *Rammathocerus brasiliensis *without B and with 1B chromosome**. (a-f) Metaphases I, (g) late diplotene and (h) diacinesis; (a) 18S rDNA; (b, e, g) 5S rDNA; (c, f, h) H3 histone; (d) DAPI only. In (c) is showed the H3 histone location in one individual from Surubim population and in the inserts in (h) are shown the hybridization patterns of 5S rDNA (red signals) and H3 (green signals) in the X and B chromosomes by two color FISH obtained from a Metaphase I. The smallest chromosome S11 that does not harbor 5S/H3 sites is indicated in (a, b, d, e, f). The X and B chromosomes, and chromosomes bearing specific hybridization (for 18S rDNA and H3 histone) signals are also indicated. Note the dispersion and co-localization of 5S rDNA and H3 histone sites, and the co-localization of 18S and 5S rDNA in three chromosomes. The meiotic plates in (g, h) are incomplete, lacking autosomes, but the X and B chromosomes are easily recognizable by their condensation patterns and univalent behavior. Chromosomes are counterstained with DAPI and the group of images (a, b) and (d, e, f) are the same metaphases submitted to two color FISH. Each bar = 5 μm.

The *in situ *hybridization of the 18S rDNA probe revealed that this sequence was located in the pericentromeric region of three autosomal chromosomes (M4, M6 and S9) in all populations analyzed (Figure 1a). The 5S rDNA was located in the pericentromeric region of all chromosomes of complement, including the X, except in the smallest autosome (S11) (Figure 1b). The dual-FISH indicated co-localization of 5S and 18S rRNA genes in the pairs M4, M6 and S9 (Figure 1a, b).

The H3 histone gene was only observed in the pair M7 in one individual from Surubim (Figure 1c), while for the other individuals from this population and for the other two populations studied this gene family presented clusters in all chromosomes, except the S11. Two color FISH using 5S rRNA and H3 histone genes as probes confirmed the patterns obtained by single hybridization revealing loci dispersion for these sequences, being co-located in almost all chromosomes, except in the pair S11, that did not presented signals for the two genes (Figure 1e, f). The FISH for 5S rRNA and H3 histone genes in individuals with 1B revealed the presence of these genes in the B chromosome (Figure 1g, h). The double-FISH (5S rDNA and H3 histone gene) was performed, revealing co-localization for these genes in the centromeric region of the B element (Figure 1h-inserts).

## Discussion

### Standard chromosomal complement

The number of sites for 18S rDNA observed in the three populations of *R. brasiliensis *is coincident with the results published by Loreto et al. [10,18] for this species. The chromosomal mapping of 45S rDNA in grasshoppers has revealed an intense variability related to number and chromosomal location of this gene cluster. According to Cabrero and Camacho [6] the variability of rDNA in grasshoppers is caused probably by three mechanisms, structural chromosome rearrangements, ectopic recombination and transpositions. Similar mechanisms were also proposed for the 45 rDNA dispersion in other insects, as Scarabaeinae beetles and Lepidoptera representatives [19,20]. For *R. brasiliensis *the most plausible explanation is the transposition or occurrence of ectopic-recombination of major rDNA sites, since this species maintains the ancestral diploid number for Acrididae, and no evident macro-chromosomal rearrangement was observed in its karyotype. On the other hand, for other Gomphocerinae the action of large chromosomal rearrangements were important in changing of rDNA location, as described for species with 2n = 17,X0 [6].

The mapping of 5S rDNA in grasshoppers was performed in about 34 species, including four Proscopiidae and some Acrididae, being the number and chromosomal location conserved in the former and variable in the later [4,5,8-10]. In *R. brasiliensis *besides the spreading of major rDNA sites it was also observed a more intense dispersion for the 5S rDNA, for almost all chromosomes of the complement. High variability for 5S rDNA cluster number was also recently observed for other Acrididae species [5]. In this work the number of 5S rDNA sites observed in this species for all populations was distinct of the data previously described by Loreto et al. [10] that described 5S rDNA sites in the bivalents L3, M5, S11 and in the B chromosome. Our result points to a divergent condition of 5S rDNA organization and dispersion among the distinct populations of this species. The differential spreading for this sequence could be explained by differences in appropriate molecular mechanisms for 5S rDNA dispersion in the distinct populations, as proposed for major rDNA in grasshoppers as a whole [6]. The ability of major rDNA clusters to move and vary in number was first observed in *Allium *[21], and recently some additional evidence has accumulated concerning the ability of rDNA to move within the genome. Some studies have proposed that transposable elements are a potential source for the movement of rDNAs [22-24].

FISH using H3 histone sequence as probe also revealed dispersion for this gene sequence, being the pattern coincident to the same location of 5S rDNA, confirmed by two color FISH. In 35 species of Acrididae and four species belonging to Proscopiidae grasshoppers the number and location of histone genes were extremely conserved, with the presence of only one site [7,8]. Cabrero et al. [7] suggested that strong purifying selection could be operating in the non-spreading of histones in Acrididae grasshopper genomes, and Cabral-de-Mello et al. [8] by the analysis of four ancient grasshoppers (Proscopiidae) proposed that the genomic association of histones and 5S rRNA genes could be ancient for grasshoppers as a whole. Bearing in mind these hypotheses, it can be proposed a posterior dispersion of H3 gene in *R. brasiliensis*, being the ancestral characteristic the occurrence of only one site that occasionally was observed in the population sampled from Surubim. The spreading of H3 histone can be directly associated to the dispersion of 5S rDNA, indicating a linked organization of these two multigene families in the genome of *R. brasiliensis*, which causes the same evolutionary patterns for the two sequences. On the contrary, the analysis of other 29 Acrididae species revealed a distinct scenario, mainly without association of 5S rDNA/H3 histone sequences, being these elements co-located only in five species, but with non co-dispersion in species with 5S rDNA spreading (see Table 1). The linked organization of 5S rRNA and histone genes was observed for other invertebrate species, by molecular approach in one mussel [25] and two crustaceans [26,27]. Moreover by chromosomal analysis Cabral-de-Mello et al. [8,16,19] have proposed the association of these sequences in the genomes of insect representatives, such as in beetles and in four ancient grasshoppers (Proscopiidae), including the observation of co-dispersion. Further molecular analysis needs to be performed in *R. brasiliensis *and in other insects to confirm the genomic nature of the association of 5S rRNA and histone H3 genes.

**Table 1:** Grasshopper species with chromosomal co-localization of 5S rRNA and H3 histone genes.

Family Subfamily	Species	Chromosome no. (in order of decreasing size)													Reference
			
		1	2	3	4	5	6	7	8	9	10	11	X	B	
Proscopiidae															
Proscopiinae	*Stiphra robusta*				p					-	-	-		-	8
	*Scleratoscopia protopeirae*				p					-	-	-		-	8
	*Scleratoscopia spinosa*				p					-	-	-		-	8
	*Tetanorhynchus silvae*				i					-	-	-		-	8
Acrididae															
Calliptaminae	*Calliptamus barbarus*		*	*		*		*	i	*	*	*	*	-	5
Eyprepocnemidinae	*Heteracris adpersa*								i					-	5
Gomphocerinae	*Rhammatocerus brasiliensis*	p	p	p	p	p	p	p	p	p	p		p	p	This work
Oedipodinae	*Aiolopus strepens*			*					i		*			-	5
	*Oedipoda charpentieri*			*	*				i					-	5
	*Oedipoda coerulescens*								i					-	5

The specific positions of co-locations are indicated by (p) proximal or (i) interstitial.

Hyphens correspond to absent chromosomes in the species with 2n = 19 and species in which animals with B chromosome were not studied for these markers or in which this element was never observed. Asterisks indicate additional sites of 5S rDNA that are not co-located with H3 histone genes for some species.

Besides the co-localization of 5S rDNA and H3 histone sequences it was also observed a similar pattern for 18S and 5S rDNA in the chromosomal pairs M4, M6 and S9 of all populations. Loreto et al. [10] described these two ribosomal genes located in distinct chromosomes of *R. brasiliensis*, being identical the position of 18S rDNA observed here. Considering that the location of 18S rDNA is stable among the populations of *R. brasiliensis*, the co-location of ribosomal elements in the three studied populations might be explained by invasion of 18S rDNA or a near region by the 5S rRNA genes through transposition events. The movement of 5S rDNA through transposition has been documented for example in mammals [28] and fish [24]. Association/co-localization of 5S and major rRNA genes was also described in other invertebrates, such as annelids, mollusks and crustaceans and also in fungi [29-31]. On the other hand, in *R. brasiliensis *there is no evidence of interspersion of 18S rDNA and H3 histones genes, considering that the distribution in the histone gene clusters is not followed by dispersion of 18S genes.

### The B chromosome

The B chromosome of *R. brasiliensis *is widely distributed among all population analyzed until the present time. Moreover a possible autosomal origin for this element using *in situ *hybridization for 5S and 45S rDNAs was proposed [10]. This conclusion was based on the presence of 5S rDNA sites restricted to autosomes and B element while absent in the X chromosome. On the contrary, our results cannot evidence an autosomal origin of the B chromosome in *R. brasiliensis *due the presence of 5S rDNA sites in almost all autosomes and also in the X chromosome, being this chromosomal marker non informative for B origin in this species. An alternative explanation can be the multiple origin of the B element in the distinct populations of *R. brasisiensis *studied with distinct conditions of 5S rDNA location. Multiregional origin for B chromosomes in grasshoppers was also previously proposed by Cabrero et al. [4] in *E. plorans*, using the mapping of rDNAs and a satellite DNA with 180 bp repeat sequences. Another limitation of the use of 5S rDNA sequence as an informative marker for definition of B origin and evolution is related to the intense evolutionary dynamics of this sequence in grasshopper genomes, as described by Cabral-de-Mello et al. [5]. In fact the presence of 5S rDNA in the B chromosome of *R. brasiliensis *could be a characteristic generated by transposition after the origin of this chromosome, and not a characteristic inherited from the chromosome responsible for its origin.

Besides the presence of 5S rDNA in B chromosomes of grasshoppers, satellite DNAs and 45S rDNA were also identified in this genomic element, as for example, in *E. plorans *and in some other animal groups, such as fish and mammals [4,32-34]. On the other hand, the presence of histone genes in B chromosomes was described only in one species until now, *L. migratoria*, being a good marker for discrimination of ancestry of this chromosome in this species [9]. The presence of H3 histone gene in the B chromosome of *R. brasiliensis *is the second case for grasshopper and for animal as a whole, indicating that in fact these chromosomes can harbor more gene sequences than was observed until now. Moreover, due the clustered organization of histone genes they can be a useful marker in the investigating of B origin. On the other hand, in the case of *R. brasiliensis*, this sequence, as well as the 5S rDNA, was not informative due their high dispersion. The presence of H3 gene in the B chromosome can be also directly associated with the dispersion of 5S rDNA, reinforcing the co-dispersion/co-localization of these two multigene families.

## Conclusion

The results presented here reinforce the hypothesis of co-localization/association of the 5S rDNA and H3 histone genes in some insect groups, and other invertebrates. This association led to the same patterns of chromosomal dispersion of these two multigene families in the genome, including both A complement and B chromosomes. These results also contribute to a better knowledge about B chromosomes content in animal kingdom. Moreover, we bring attention in the definition of chromosomal markers to be applied for analysis of B origin, depending on the mechanisms of evolution for the chosen sequence in the genomes to be investigated. Specifically for the case of *R. brasiliensis *the ribosomal DNAs and histone genes apparently are not sufficient for definition of B origin, due their intense evolutionary dynamics leading to spread of clusters in the genome and remarkable differences among population of the species.

## Competing interests

The authors declare that they have no competing interests.

## Authors' contributions

NLO and DCCM carried out the conventional and molecular cytogenetic analysis and draft the manuscript. VL and MFR helped in the discussion of the obtained data. RCM, CM and DCCM conceived of the work, and participated in its design, drafted and revised the manuscript. All authors analyzed all results and read and approved the final manuscript. DCCM revised the manuscript with the suggestions from the reviewers.

## References

[B1] Long EO Dawid IB Alternative pathway in processing of ribosomal RNA precursor in *Drosophila melanogaster* J Mol Biol 1980 138 373 378 10.1016/0022-2836(80)90070-4 6774101

[B2] Maxson R Cohn R Kedes L Mohun T Expression and organization of histone genes Annu Rev Genetics 1983 17 239 377 10.1146/annurev.ge.17.120183.001323 6421226

[B3] Nei M Rooney AP Concerted and birth-and-death evolution of multigene families Annu Rev Genetics 2005 39 121 152 10.1146/annurev.genet.39.073003.112240 PMC1464479 16285855

[B4] Cabrero J Bakkali M Bugrov A Warchalosk-Sliwa E López-León MD Perfectti F Camacho JPM Multiregional origin of B chromosome in the grasshopper *Eyprepocnemis plorans* Chromosoma 2003 112 207 211 10.1007/s00412-003-0264-2 14628147

[B5] Cabral-de-Mello DC Cabrero J López-León MD Camacho JPM Evolutionary dynamics of 5S rDNA location in acridid grasshoppers and its relationship with H3 histone gene and 45S rDNA location Genetica 2011 139 921 931 10.1007/s10709-011-9596-7 21755328

[B6] Cabrero J Camacho JPM Location and expression of ribossomal RNA genes in the grasshopper: abundance of silent and cryptic loci Chromosome Res 2008 16 595 607 10.1007/s10577-008-1214-x 18431681

[B7] Cabrero J López-León M D Teruel M Camacho JPM Chromosome mapping of H3 and H4 histone gene clusters in 35 species of acridid grasshopper Chromosome Res 2009 17 397 404 10.1007/s10577-009-9030-5 19337846

[B8] Cabral-de-Mello DC Martins C Souza MJ Moura RC Cytogenetic mapping of 5S and 18 rRNAs and H3 histone genes in 4 ancient Proscopiidae grasshopper species: contribution to understanding the evolutionary dynamics of multigene families Cytogenet Genome Res 2011 132 89 93 10.1159/000317476 20668370

[B9] Teruel M Cabrero J Perfectti F Camacho JP B chromosome ancestry revealed by histone genes in the migratory locust Chromosoma 2010 119 217 225 10.1007/s00412-009-0251-3 20016909

[B10] Loreto V Cabrero J López-León MD Camacho JPM Souza MJ Possible autosomal origin of macro B chromosomes in two grasshopper species Chromosome Res 2008 16 233 241 10.1007/s10577-007-1188-0 18095175

[B11] Camacho JPM Sharbel TF Beukeboom LW B-Chromosome evolution Phil Trans R Soc Lond B 2000 355 163 178 10.1098/rstb.2000.0556 PMC1692730 10724453

[B12] Camacho JPM Ryan T Gregory B Chromosomes Evolution of the genome 2005 London: Academic Press 223 286

[B13] López-León MD Cabrero J Dzyubenko VV Bugrov AG Karamysheva TV Rubtsov NB Camacho JPM Differences in ribosomal DNA distribution on A and B chromosomes between eastern and western population of the grasshopper *Eyprepocnemis plorans plorans* Cytogenet Genome Res 2008 121 260 265 10.1159/000138894 18758168

[B14] Teruel M Cabrero J Perfectti F Camacho JP Quantitative analysis of NOR expression in a B chromosome of the grasshopper *Eyprepocnemis plorans* Chromosoma 2009 118 291 301 10.1007/s00412-008-0197-x 19048264

[B15] Sambrook J Russel DW Molecular cloning: A laboratory manual 2001 3 New York: Cold Spring Harbor Laboratory Press

[B16] Cabral-de-Mello DC Moura RC Martins C Chromosomal mapping of repetitive DNAs in the beetle *Dichotomius geminatus *provides the first evidence for an association of 5S rRNA and histone H3 genes in insects, and repetitive DNA similarity between the B chromosome and A complement Heredity 2010 104 393 400 10.1038/hdy.2009.126 19756039

[B17] Levan A Fredga K Sandberg AA Nomenclature for centromeric position on chromosomes Hereditas 1964 52 201 220

[B18] Loreto V Cabrero J López-León MD Camacho JPM Souza MJ Comparative analysis of rDNA location in five Neotropical gomphocerine grasshopper species Genetica 2008 132 95 101 17486415 10.1007/s10709-007-9152-7

[B19] Cabral-de-Mello DC Oliveira SG Moura RC Martins C Chromosomal organization of the 18S and 5S rRNAs and histone H3 genes in Scarabaeinae coleopterans: insights into the evolutionary dynamics of multigene families and heterochromatin BMC Genetics 2011 12 88 21999519 10.1186/1471-2156-12-88 PMC3209441

[B20] Nguyen P Sahara K Yoshido A Marec F Evolutionary dynamics of rDNA clusters on chromosomes of moths and butterflies (Lepidoptera) Genetica 2010 138 343 354 10.1007/s10709-009-9424-5 19921441

[B21] Schubert I Mobile nucleolus organizing regions (NORs) in *Allium *(Liliaceae S- Lat)- inferences from the specifity of silver staining Plant Syst Evol 1984 144 291 305 10.1007/BF00984139

[B22] Raskina O Barber JC Nevo E Belyayev A Repetitive DNA and chromosomal rearrangements: speciation-related events in plant genomes Cytogenet Genome Res 2008 120 351 357 10.1159/000121084 18504364

[B23] Zhang X Eickbush MT Eickbush TH Role of recombination in the long-term retention of transposable elements in rRNA gene loci Genetics 2008 180 1617 1626 10.1534/genetics.108.093716 18791229 PMC2581962

[B24] Silva M Matoso DA Vicari MR Almeida MC Margarido VP Artoni RF Physical mapping of 5S rDNA in two species of knifefishes: *Gymnotus pantanal *and *Gymnotus paraguensis *(Gymnotiformes) Cytogenet Genome Res 2011 134 303 307 10.1159/000328998 21654160

[B25] Eirín-López JM Ruiz MF González-Tizón AM Martínez A Sánchez L Méndez J Molecular evolutionary characterization of the mussel *Mytilus *histone multigene family: first record of a tandemly repeated unit of five histone genes containing an H1 subtype with ''orphon'' features J Mol Evol 2004 58 131 144 10.1007/s00239-003-2531-5 15042333

[B26] Andrews MT Vaughn JC Perry BA Bagshaw C Interspersion of histone and 5S RNA genes in *Artemia* Gene 1987 51 6 l 67 10.1016/0378-1119(87)90474-4 3596239

[B27] Barzotti R Pelliccia F Bucciarelli E Rocchi A Organization, nucleotide sequence, and chromosomal mapping of a tandemly repeated unit containing the four core histone genes and a 5S rRNA gene in an isopod crustacean species Genome 2000 43 341 345 10.1139/g99-142 10791823

[B28] Drouin D Expressed retrotransposed 5S rRNA genes in the mouse and rat genomes Genome 2000 43 213 215 10.1139/g99-100 10701135

[B29] Drouin G Moniz de Sá M The concerted evolution of 5S ribosomal genes linked to the repeated units of other multigene families Mol Biol Evol 1995 12 481 493 7739390 10.1093/oxfordjournals.molbev.a040223

[B30] Vitturi R Colomba M Mandrioli M Pirrone AM rDNA (18S-28S and 5S) co-localization and linkage between ribosomal genes and (TTAGGG)*_n _*telomeric sequence in the earthworm *Octodrilus complanatus *(Annelida: Oligochaeta: Lumbricidae) revealed by single- and double-colour FISH J Hered 2002 93 279 282 10.1093/jhered/93.4.279 12407215

[B31] Vitturi R Sineo L Volpe N Lannino A Colomba M Repetitive DNAs in the slung *Milax nigricans*: association of ribosomal (18S-28S and 5S rDNA) and (TTAGGG)*_n _*in telomeric sequences in slung *M. nigricans *(Mollusca: Gastropoda: Pulmonata) Micron 2004 35 255 260 10.1016/j.micron.2003.11.011 15003612

[B32] Stitou S Díaz de La Guardia R Jiménez R Burgos M Inactive Ribosomal cistrons are spread through the B chromosome *Rattus rattu*s (Rodentia, Muridae). Implications for their origin and evolution Chromosome Res 2000 8 305 311 10.1023/A:1009227427575 10919721

[B33] Silva MJ Yonenaga-Yassuda Y B chromosome in Brazilian rodents Cytogenet Genome Res 2004 106 257 263 10.1159/000079296 15292600

[B34] Poletto AB Ferreira IA Martins C The B chromosome of the cichlid fish *Haplochromis obliquidens *harbors 18S rRNA genes BMC Genetics 2010 11 1 20051104 10.1186/1471-2156-11-1 PMC2806386

